# Bioinks in systems biology: integrating biomaterial design with cellular network modelling for predictive biofabrication

**DOI:** 10.3389/fbioe.2026.1854783

**Published:** 2026-06-30

**Authors:** Inamul Hasan Madar, Jayant Pawar, Punitha Muruganantham, Syed Suhaib Ahmed, Vansh Vohra, Janakiraman Velayudam

**Affiliations:** 1 Center for Integrative Omics Data Science (CIODS), Yenepoya (Deemed to be University), Mangalore, Karnataka, India; 2 Krishna Institute of Science and Technology, Krishna Vishwa Vidyapeeth (Deemed to be University), Taluka-Karad, Maharashtra, India; 3 Department of Pharmaceutics, Yenepoya Pharmacy College and Research Centre, Yenepoya (Deemed to be University), Mangalore, Karnataka, India; 4 M.M College of Pharmacy, Maharishi Markandeshwar (Deemed to be University), Mullana, Haryana, India

**Keywords:** biofabrication, bioink, mechanistic modeling, multi-omics, systems biology

## Abstract

Bioinks facilitate patient-relevant tissue models by merging living cells with a programmable matrix whose composition, mechanics, and transport properties can be altered in three dimensions and tracked over time. Modern bioink formulations use nano and micro technologies that function as real-time biosensors of the cellular microenvironment. This narrative review offers systems biology and artificial intelligence perspectives crucial for bioink design due to its established biomaterial role, which acts as both a passive structural support and an active regulator in complex biological networks. Additionally, we briefly discuss current challenges and future perspectives on using bioinks as behaviour in various aspects that offers real time monitoring, and linking material composition to biological performance outcomes.

## 1 Introduction

Bioinks should be treated as time-varying perturbation systems rather than inert carriers; their chemistry and mechanics can change during culture or after implantation, thereby acting as controllable inputs that reshape cell-state trajectories. In 4D bioprinting, stimuli-responsive bioinks are engineered to alter structure or function in response to cues such as pH, electric fields, or magnetic fields, enabling programmed, time-dependent changes in stiffness, ligand accessibility, degradation, and solute transport the same variables that gate mechanotransduction and downstream signaling. This responsiveness enables practical delivery functions (e.g., temporally staged factor release) and dynamic microenvironment control needed for processes such as vascular stabilization and maturation, moving bioinks from “support materials” to active regulators of tissue formation and therapeutic delivery (Fatimi et al., 2022).

Reductionist bioink screening prioritizes isolated, short-horizon metrics (processability/printability, initial stiffness, acute post-print viability) that do not reliably predict tissue maturation or function. “Printability” is frequently ill-defined conflating extrusion processability with post-print geometric fidelity and is often inferred from limited rheological proxies without agreed success criteria or accounting for nozzle geometry, deposition dynamics, and substrate interactions. This narrow optimization induces predictable trade-offs: increasing viscosity or stiffness to improve shape fidelity typically raises extrusion shear stresses, compromising viability and proliferative capacity within the printable window (Kyle et al., 2018; Gillispie et al., 2020; Ullah et al., 2025). Reporting short-term survival does not address dominant long-term failure modes restricted nutrient transport, delayed/absent vascularization, and degradation kinetics mismatched to matrix remodeling that determine phenotype stability and functional integration (Ullah et al., 2025). However, formulations that score well on proxy print metrics may still fail to support sustained differentiation, ECM deposition, and *in vivo* performance because the evaluation framework is not anchored to multi-outcome, time-resolved biological endpoints. The practical requirement is therefore a multi-parametric, multi-objective evaluation that links fabrication constraints to long-term biological performance and generates datasets suitable for mechanistic and predictive modeling (Kyle et al., 2018; Gillispie et al., 2020; Ullah et al., 2025).

Bioinks enable patient-relevant tissue models by combining living cells with a programmable matrix whose composition, mechanics, and transport properties can be controlled in three dimensions and tracked over time. Bioprinting has been used to assemble constructs incorporating patient-derived tumor and stromal compartments together with defined ECM and growth-factor cues to approximate key features of the tumor microenvironment that are poorly captured by 2D systems and often distorted in animal models, thereby improving the significance of drug response studies (Kyle et al., 2018; Fonseca et al., 2020). Hydrogel bioinks (e.g., alginate, gelatin, fibrinogen, and GelMA-based systems) provide tunable adhesion, stiffness, and remodeling capacity while meeting fabrication constraints for spatially resolved patterning (Liu and Jin, 2025). Patient-derived iPSC platforms further enable genotype-controlled disease modeling through isogenic comparisons, strengthening causal inference for developmental and disease mechanisms (Fonseca et al., 2020). Advanced fabrication modalities (including laser-assisted printing and microfluidic integration) matter here only insofar as they increase control over spatial interfaces, gradients, and vascular patterning, which are dominant determinants of cell-state heterogeneity and function (Liu and Jin, 2025; Robazzi et al., 2026).

AI/ML approaches can accelerate bioink development by learning mappings between formulation/process variables and outcomes such as shape fidelity, viability, and degradation kinetics, and by enabling parameter optimization and, increasingly, closed-loop control but only when trained on standardized, multi-modal, model-ready datasets (Robazzi et al., 2026). Despite progress, routine clinical deployment remains constrained by the core engineering biology coupling problem: achieving fabrication robustness without sacrificing long-term function, validating predictive models across cell sources, and navigating regulatory characterization for combination products (Fonseca et al., 2020; Liu and Jin, 2025). The major technical challenges associated with AI/ML-assisted predictive biofabrication and the quantitative relationships between bioink properties, mechanotransduction pathways, and biological outcomes are summarized in Tables 1 and 2.

**TABLE 1:** Technical challenges and considerations in AI/ML-assisted predictive biofabrication.

Challenge	Impact	Possible solution
Small datasets	Overfitting	Data augmentation, transfer learning
Batch effects	Reduced reproducibility	Dataset harmonization
Heterogeneous modalities	Integration difficulty	Multimodal architectures
Black-box models	Poor interpretability	Explainable AI
Limited validation	Weak generalizability	External benchmarking
Inter-lab variability	Reduced robustness	Standardized protocols

**TABLE 2:** Quantitative relationships between bioink properties, mechanotransduction pathways, and biological outcomes.

Bioink parameter	Quantitative range	Dominant pathway	Biological outcome
Soft stiffness	∼1–5 kPa	Reduced YAP/TAZ, low RhoA/ROCK	Neurogenic differentiation, M2 macrophages
Intermediate stiffness	∼10–30 kPa	Integrin/FAK activation	Endothelial maturation, vascular stabilization
High stiffness	>40–100 kPa	YAP/TAZ, ROCK, FAK	Osteogenesis, matrix mineralization
Rapid stress relaxation	Fast viscoelastic remodeling	Cytoskeletal remodeling	Increased spreading and differentiation
High ligand density	Increased integrin occupancy	FAK/MAPK signaling	Enhanced adhesion and proliferation
Fast degradation	Short remodeling half-life	Dynamic signaling turnover	Vascular infiltration
Slow degradation	Sustained matrix signaling	Persistent focal adhesions	Structural maturation

## 2 Bioinks as multi-modal data generators

**FIGURE 1:**
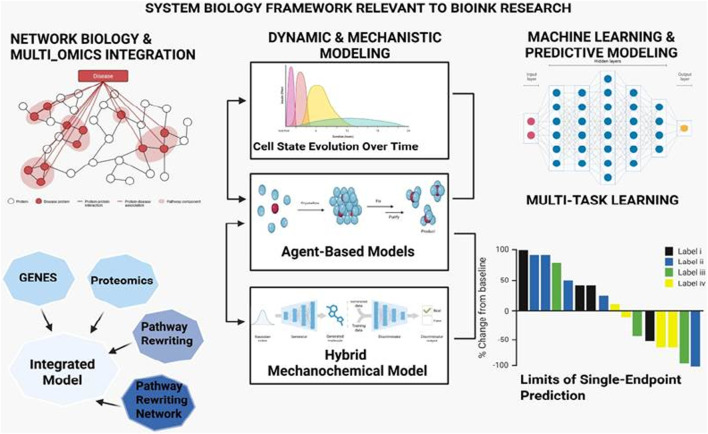
Systems biology framework relevant to bioink research. Systems biology methods combine multi-omics data, mechanistic models and machine learning systems to learn and predict cellular responses in biofabricated systems. Network biology and multi-omics integration is the integration of genomic, transcriptomic, proteomic, and pathway scale information to determine signaling interactions and pathway rewiring events induced by bioink-mediated perturbations. Mechanistic and dynamic models, such as ordinary differential equation (ODE)-based systems, agent-based models, and hybrid mechanochemical models, can be used to simulate time-dependent cell states and emergent tissue dynamics. Predictive modeling and machine learning methods (especially multi-task learning strategies) can be used to predict more than one biological endpoint and address the drawbacks of single-endpoint prediction. Together, these systems-level strategies enable predictive biofabrication by using combined analysis of the biomaterial properties, cellular signaling, and tissue-level responses.

Bioinks are multi-modal data generators in the context of predictive biofabrication, and can provide indispensable physical, chemical, and biological information to cellular network modeling (Figure 1). These materials are not considered as passive scaffolds anymore; they are active members of a digitally coordinated, feedback loop of data (Moroni et al., 2018). Bioinks give important mechanical data, such as viscosity, shear-thinning coefficients, and compressive moduli, which are crucial in predicting structural performance (Cruz et al., 2023). These parameters are then used by machine learning algorithms in order to identify non-intuitive correlations between material design and end print roughness or shape fidelity. An example is in high-throughput systems that are now used to record real-time rheological drift in order to maintain stringent statistical process control (Ning et al., 2023).

Recent bioink formulations incorporate micro- and nanotechnologies which serve as real time biosensors of the cellular microenvironment (Maan et al., 2022).

- Imaging data: Nanoparticles that have been used as contrast reagents make it possible to monitor scaffold placement and degradation non-destructively in real-time using either computed tomography (CT) or magnetic resonance imaging (MRI) (Rasouli et al., 2025).
- Physiological sensing: Smart bioinks can incorporate optical sensor nanoparticles to image oxygen or sensors to measure temporal variations in pH and electrical conductivity.
- Mechanical phenotyping: Bioprinted microarchitectures can be measured by mechanical strain devices that can measure tissue morphogenesis and contractility throughout culture (Lind et al., 2017).

The biofabricated constructs produce more clinically relevant complex biological datasets as compared to the traditional ones (Neufeld et al., 2021).

- Transcriptomic and genomic data: RNA sequencing (RNA2-seq) of bioprinted models reveals significant gene expression patterns that are highly predictive of *in vivo* phenotypes, which provides systems biology models with high-fidelity information.
- High-Throughput Screening (HTS): Bioprinted arrays generate large-scale amounts of pharmacological data, with deep learning models categorizing cellular phenotypes and drug responses in disease models, including glioblastoma or Huntington disease.
- Multi-Mode Systems Integration: HTBF systems integrate microfluidic sensors and AI feedback loops to coordinate the management of fabrication and maturation stages of data.

### 2.1 Classes of bioinks and their biological perturbation profiles

In the context of bioprinting, bioinks are categorized in terms of their material origin, and the mechanistic clues they provide to the cellular microenvironment; each of these classes is defined by a distinctive pattern of biological perturbation.

Among them, the natural polymers, gelatin methacrylate (GelMA), fibrinogen, and collagen, with intrinsic Arg 2-Gly 2-Asp (RGD) motifs promoting cell adhesion, proliferation, and remodeling of a matrix are fundamental (Zhu et al., 2019). Such substrates are mechanically controllable: e.g., tuning the gelatin concentration of fibrin-based inks provides a reliable recapitulation of cerebral tissue stiffness, and thus controls glioblastoma proliferation dynamics and invasion (Neufeld et al., 2021). In comparison, synthetic polymers like Pluronic 127 or polyethylene glycol (PEG) are commonly used as sacrificial scaffolds or structural supports, but contain no or few bioactive ligands of natural matrices (Raees et al., 2023).

The decellularized extracellular matrix (dECM) inks provide extremely precise biochemical signals due to the maintenance of the original tissue niche, however, the concentration of the inks presents significant effects on cellular behavior (Abaci et al., 2023). The 3% w/v dosage of dECM prepared based on the amniotic membrane has been established to inhibit the growth of fibroblasts and promote a rounded phenotype, and the 2% w/v concentration promotes a spindle-like form and stimulates the proliferation (Kafili et al., 2024).

Composite inks combine micro-/nanofillers (0D, 1D and 2D) in order to enhance mechanical performance and add stimuli responsive features (Rasouli et al., 2025). As an example, incorporation of electrically conductive gold nanorods or nanowires disrupts cellular organization by orienting electrosensitive cells including cardiomyocytes and myoblasts. Smart composites go even further by integrating microspheres that carry drugs to provide growth factors thus inducing the neural progenitor to form dopaminergic or glial lineages in a three dimensional structure (Richards et al., 2013).

Specific additives are used in order to tune the biological readout of the end structure. PRP incorporated in alginate-gelatin implants enhances cellular activity and induces angiogenesis by the long-term release of autologous growth factors. Similarly, the addition of extracellular matrix components, including Geltrex, is essential in preserving the stemness and viability of human neuro-progenitor cells in GelMA-based solutions, and new materials are now being explored to deliver lipidic motifs, such as cholesteryl ester liquid crystals (CELC), to improve cell adhesion as well as to mimic the elasticity of native membranes (Zhao et al., 2016).

Natural polymers such as GelMA can be used to regulate genes by retaining neural progenitor stemness but significantly decreasing the level of NANOG (Malekpour and Chen, 2022). The scaffolds disrupt signaling pathways *via* mechanotransduction; the rigidity of substrates directly determines neurite elongation and directs stem cell lineage commitment. The softness of material is preferred to enable metabolic flux in these constructs since the constructs can easily provide better diffusion of oxygen and nutrients compared to overly rigid matrices (Li et al., 2021). The fineness of the tuning of mechanic can be done through cross-linking density and polymer concentration wherein, the more the degree of methacrylation, the higher the matrix modulus, but the inhibition of the cellular networks formation (Abdulmaged et al., 2022; Cruz et al., 2023). The systems-level mechanotransduction pathways underlying these material-induced cellular responses are discussed in greater detail in Sections 3.1 and 4.1.

To provide biochemical signals, tissue-specific dECM inks feed biomaterials that disrupt gene regulation by inducing myogenic markers in muscle constructs. They improve cell-matrix communications by maintaining the intrinsic biochemical niche, and outperform the simple hydrogel systems. Introduction of changes in dECM concentration can be used to control metabolic production through changes in mass transfer and nutrient accessibility in the 3D architecture. Mechanically, dECM inks are preserved to mimic the biophysical characteristics of target organs giving the specific moduli needed to differentiate healthy and fibrotic cardiac tissue (Soon et al., 2014; Kafili et al., 2024).

Nanomaterials are used in composite and smart inks to strategically perturb gene regulation; electroconductive fillers, and carbon nanotubes, which are used to increase cardiac expression of genes, including Tnnt2 and Nkx2-5 (Aizarna-Lopetegui et al., 2023). These are smart formulations that regulate signaling in both magnetic and electric circuits, which provide external structural information to orient electrosensitive cardiomyocytes and myoblasts (Chen et al., 2019). Gold nanorods or black phosphorus nanosheets impregnated into hybrid hydrogels maintain high cellular metabolism through the stable and conductive microenvironment delivery to cultured cells. Inorganic fillers like hydroxyapatite and nanosilicates reinforce mechanics significantly to increase mechanical strength and stiffness to resemble native load bearing tissues (Chimene et al., 2018).

**FIGURE 2:**
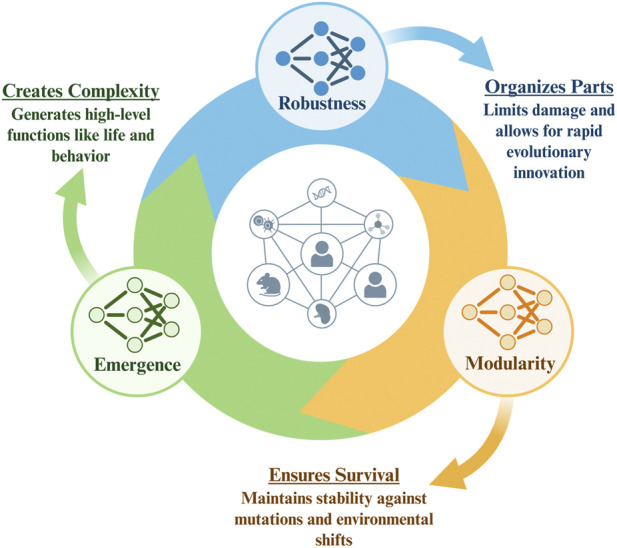
Core systems biology principles underlying predictive biofabrication. The systems biology paradigms explain that biological behavior is an emergent property of interacting multi-scale systems, and not a property of individual molecular parts. Three key principles of systems-level behavior that apply to biofabrication are demonstrated: (i) emergence, wherein interactions between molecular, cellular, and tissue-level components form higher-order biological functions; (ii) robustness, which allows biological systems to sustain functional stability in response to environmental perturbation, material variability, or signaling fluctuations; and (iii) modularity, where semi-independent biological subnetworks organize complex biological processes and allow adaptive responses and evolutionary plasticity. Collectively, these principles offer the conceptual basis of combining bioink-mediated perturbations and systems biology, multi-omics analysis, and predictive biofabrication frameworks.

The bioactive additives like PRP influence the regulation of genes by polarizing the macrophages to an anti-inflammatory M2-type state, which exhibits a reduction in iNOS and increment of Arg-1 gene expression (Anitua et al., 2024). These additives mediate signaling and serve as reservoirs of growth factors and cytokines, and thus promote complex cell-environment interaction. New lipid-based additives such as CELC aid in metabolism stabilizing bilayer membranes and modeling *in vivo* membrane conditions (Nasajpour et al., 2020). Alterations at the physical interface include the use of liquid crystals to rough the surfaces to allow cells to attach to the surface and PRP to improve tensile strength of the scaffold built (Zhao et al., 2022). The core systems biology principles relevant to predictive biofabrication are summarized in Figure 2.

### 2.2 Data modalities elicited by bioink cell interactions

The nature of bioink cell interactions creates a complex range of data modalities that cuts across metabolic, structural and biophysical space, and provides the conceptual clarity needed to predict biofabrication (Kim, 2023). These contacts take the epicentric role of a developing definition of biocompatibility, where the bioink is no longer a primitive biosafety requirement, but an imperative of biofunctionality, where the bioink must direct the actions of the desired cells and cellular differentiation, e.g., adhesion, growth and differentiation (Kyle et al., 2017).

The normal forms of metabolic and viability data are commonly the primary measure of bioink functionality; standardized tests, including MTT, MTS and AlamarBlue, can be used to measure mitochondrial activity in three-dimensional structures. Similarly, the membrane integrity tests, especially live/dead fluorescence staining, can provide important spatial data on the percentage of viable cells and their distribution on the bioprinted structure (Von Recum and Laberge, 1995).

Improved imaging methods provide high-resolution structural information which can be used to carefully visualize cells which are embedded in complex 3-D scaffolds (Teodori et al., 2017). Laser confocal scanning microscopy (LCSM) provides the ability to produce three dimensional models of sub-cellular structures, e.g., nuclei, cytoskeletal filaments, and two-photon laser scanning microscopy (TPLSM) is better penetrating and less phototoxic, allowing longitudinal imaging of the cell spreading and differentiation processes deep into the bioink (Song et al., 2013). In addition to these optical approaches, scanning electron microscopy (SEM) and focussed ion beam SEM (FIB-SEM) provide ultrastructural information about the internal porosity of the bioink and the fine extension of cytoplasmic processes to the matrix around them (Chen et al., 2020).

Biophysical data modalities are being more exploited to understand the mechanical conversation between the cell and its artificially created milieu. Atomic force microscopy (AFM) is a technique that induces a quantitative response to cell-matrix adhesion forces and the local stiffness of the biomaterial on a sub-nanometric scale (Teodori et al., 2017). TMF is used in the determination of the dynamic rotational moments and mechanical forces exerted by the cells on the bioink and thus provides a window into mechanotransduction signalling pathways. Moreover, several particle tracking (MPT) microrheology is also used to describe the spatiotemporal rheological transitions of the pericellular compartment, which reflects the local remodelling of the hydrogel network by cell-secreted enzymes.

Real-time, non-destructive modalities like optical coherence tomography (OCT) provide longitudinal information regarding cellular dynamics and tissue development, without disturbing the integrity of the 3D construct. X-ray-based micro-computed tomography (microCT) and synchrotron radiation microCT (SRuCT) provide 3-D high-resolution data on mineralization events and the uniformity of cellular distribution in opaque or mineralized scaffolds. FRET biosensors have the potential to be used at the molecular level to measure the direct correlation between the ligand-receptor bond formation and subsequent phenotypic expression (Ouyang et al., 2016).

**FIGURE 3:**
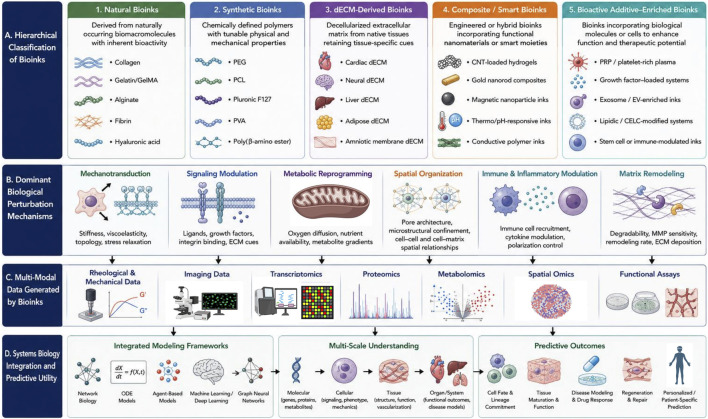
Bioinks as systems-level perturbation platforms for predictive biofabrication. Bioinks are dynamic biological perturbation systems that can tune cellular signaling, cell metabolism, cell spatial organization, immune response, and remodeling of the extracellular matrix. **(A)** Bioinks are hierarchically classified, such as natural bioinks, synthetic polymers, decellularized extracellular matrix (dECM)-based systems, composite/smart bioinks, and bioactive additive-enriched bioinks. **(B)** Overwhelming biological perturbation pathways that bioinks stimulate, such as mechanotransduction, signaling modulation, metabolic reprogramming, spatial organization, immune/inflammatory control, and matrix remodelling. **(C)** Bioink system-generated multi-modal datasets such as rheological and mechanical, imaging, transcriptomics, proteomics, metabolomics, spatial omics, functional assays. **(D)** Systems biology integration frameworks that integrate network biology, mechanistic modeling, machine learning, and graph-based methods to allow multi-scale insight into bioink-mediated cellular responses and predictive biofabrication outcomes, such as tissue maturation, disease modeling, regeneration, and personalized therapeutic prediction.

The interactions of bioinks also respond to complicated biochemical and proteomic information defining the two-way communication between cellular networks and the bioprinted environment. Microarray array cytokine profiling of multicellular models indicates the paracrine signalling loops that have been developed in multicellular models, including TGF-β1, IL-8, and VEGF expression, which governs the pathological homeostasis and angiogenesis. Lastly, the combination of these different modalities of data *in silico* predictive modelling and machine-learning strategies can optimize bioink formulations and printing parameters, and, as a result, allow biological results to be controlled precisely in a standardized fashion (Kong et al., 2006). The systems-level relationships between bioink classification, biological perturbation mechanisms, multi-modal data generation, and predictive biofabrication utility are summarized in Figure 3.

Collectively, these systems biology principles form the conceptual framework of how bioink physicochemical properties can be incorporated into the dynamic cellular network responses. The applications of computational modeling, multi-omics integration and machine learning frameworks in converting these biological interactions into predictive biofabrication strategies are discussed in the following sections.

## 3 Systems biology frameworks relevant to bioink research

Systems biology insights are crucial for bioink design due to its engineered biomaterial function, not only as passive structural support but also as an active regulator within complex biological networks. Traditionally, multidisciplinary fields such as physiology, immunology, neurobiology, and developmental biology have utilised the system-level understanding. Since the 1980s, systems biology has advanced rapidly with technological breakthroughs that enabled comprehensive system-wide analysis. Emergence of DNA sequencing made it possible to map whole-genome sequences and polymorphisms; microarray/next-generation sequencing (NGS) platforms enabled differential gene expression profiling; and development in mass spectrometry supports large-scale proteomic and metabolomic analysis. The enormous amount of data produced by these high-throughput technologies enhanced the development of computational biology and bioinformatics, providing tools to trace the flow of biological data from genes to proteins to functional cellular outcomes. These advanced techniques laid the foundation of modern systems biology, an integrative framework for interpreting how bioinks modulate biological behaviour across cellular, molecular and tissue levels.

The core concept of systems biology relies on emergence, robustness, and modularity (Whitacre, 2012) that are crucial for bioink design. Emergence denotes the system-level features that arise from interactions between constituents rather than predicting each constituent independently (Lüttge, 2012). For instance, life itself is an emergent property of harmonized interaction of DNA, proteins, lipids, and carbohydrates, which are well studied biochemical characteristics.

Interestingly, the biological cell fate choices, tissue morphogenesis, and collective behaviours (such as organoid self-assembly) falls under emergent phenomena in the setting of bioinks (Farahani et al., 2022) that lead to the dynamic interaction between the matrix constituents, soluble substances, cells and mechanical stimuli. The potential of the biological system to retain the functional behaviour in the aspect of genetic mutation, stochastic fluctuations and environmental perturbation was termed as robustness (Mukhamedova and Mukhamedov, 2025). Fascinatingly, the major foundation of robustness arises from change in the feedback loops (positive and negative) in molecular signaling and gene regulatory networks (Momin et al., 2025). On this note, the noise reduction and output stability are mediated by negative feedback mechanism, while the bistable switching (like lineage commitment) and signal amplification are regulated by positive feedback. The evaluation of engineered bioink systems depends on robustness whether it retains the inflammatory signals, nutritional gradients or mechanical stress (Tripathi et al., 2025). In-depth comprehension of feedback architecture is required to design bioinks that either purposefully disrupt (to remodel cell destiny) or maintain (to sustain stable differentiation) the network robustness.

In addition, the feature modularity distinguishes the intricate biological mechanism. A network of highly interacting nodes that perform biological functions is called a module in biology (Alcalá-Corona et al., 2021). On the other hand, engineering modules are functional components with predetermined inputs and outputs. Gene regulation, metabolic processes, and signalling cascades are examples of biological processes, which demonstrate module behaviour (Bonneau, 2008). By confining disruptions to designated segments, modularity increases robustness and prevents local failures from propagating throughout the network. Furthermore, the module wiring disruption might promote evolution (Voicu et al., 2025). Therefore, bioink design could mimic modularity through separate but interdependent components such as biochemical modules, tunable stiffness and structural modules.

By including separate but interdependent components including mechanical modules (tunable stiffness), biochemical modules (growth factor presentation), and structural modules (topology or porosity) bioink design can mimic modularity. Bioinks can be designed and linked precisely to alter certain pathways of signalling without completely destabilising cellular networks. Systems biology provides a theoretical and computational framework to comprehend bioinks as active regulators of biological networks rather than passive scaffolding. Considering emergence, robustness, and modularity can help bioink design move toward predictive, multiscale methods that better control cell behaviour, tissue formation, and long-term utility. The major systems biology components relevant to predictive biofabrication are summarized in Figure 1

### 3.1 Network biology and multi-omics integration

Multi-omics consists of integrated circuits of genomics, transcriptomics, epigenomics, proteomics, metabolomics, and related data types (Luo et al., 2024). Recently, multi-omics has grown vital to current biomedical studies as advances facilitate the simultaneous collection of multiple omics layers from the same biological samples. Although similar datasets are now expanding across neurological, renal, and hepatic disorders, integration of these datasets is still challenging because of data heterogeneity, high dimensionality, sparsity and limited sample sizes. Interestingly, The Cancer Genome Atlas (TCGA) has shown the potential of integrated multi-omics analyses in revealing disease mechanisms, particularly in cancer (Liao and Wang, 2023).

Network biology offers a structured framework for addressing these difficulties by modelling biological entities as nodes and their connections as edges (Zhang et al., 2014), which can be drawn from prior knowledge bases like the Kyoto Encyclopedia of Genes and Genomes (KEGG) or inferred computationally to reveal novel pathway associations. Network-based multi-omics integration enhances the interpretability of intricate disease-associated molecular systems and allows for a more robust representation of cross-layer biological interactions when combined with established machine learning and new graph deep learning techniques under supervised and unsupervised paradigms.

Bioinks influence cellular biology processes, including gene regulation, metabolic fluxes and protein signalling (Elango and Zamora-Ledezma, 2025). The system biology offers techniques for mapping and evaluating these layers as interconnected networks, allowing biologists to observe how perturbations (such as material cues) spread through molecular systems. The nodes (genes, proteins, and metabolites) and edges (functional linkages) of biological network models (interactomes, co-expression networks, and protein–protein contacts) reflect system-wide behaviour (Agamah et al., 2022) that goes beyond discrete molecular observations. A scaffold for interpreting how bioink environments may affect cellular states is provided by the network-based integration of multi-omics data (genomics, transcriptomics, proteomics and metabolomics), which has been shown to identify important drivers of complex phenotypes and emergent behaviours in biological systems.

For instance, multi-omics integration research has developed comprehensive maps of metabolic networks and their regulatory contexts, demonstrating the relationship between metabolite shifts and upstream genomic and proteomic signalling. This conceptual framework is specifically relevant to the assessment of how bioinks modify metabolic pathways and decisions regarding cell fate.

Stiffness, viscoelasticity, ligand presentation and degradability, are the characteristics of material cues inherent in bioinks (Wei et al., 2021) that are becoming more widely acknowledged as active modulators of intracellular signalling networks as opposed to passive structural supports. According to systems biology study, mechanotransduction pathways involving integrins, RhoA/ROCK, focal adhesion kinase (FAK)and YAP/TAZ may trigger extensive transcriptional and signalling reprogramming through extracellular matrix (ECM) mechanics, which will ultimately alter gene regulatory and metabolic networks (Mosaddad et al., 2021; Katoh, 2025). These findings lend credence to the concept of pathway rewiring, in which physical and biochemical signals produced by biomaterials reorganise network connectivity and alter information flow across gene-protein-metabolite layers. It has become increasingly evident through experimental evidence that certain bioink physicochemical properties are quantitative regulators of mechanotransduction pathways and resultant cellular phenotypes. One of the most well-validated parameters that regulate pathway rewiring is matrix stiffness, which can be regulated by integrin-mediated focal adhesion maturation and cytoskeletal tension. Soft matrices with a range of 1–5 kPa have been demonstrated to repress YAP/TAZ nuclear localization and to promote neurogenic differentiation and pro-regenerative macrophage polarization, whereas higher stiffness substrates over 40,100 kPa can augment RhoA/ROCK activation, focal adhesion kinase (FAK) signaling, and osteogenic transcriptional programs such as RUNX2 and ALP expression (Engler et al., 2006; Chaudhuri et al., 2020). Mediating stiffnesses (between 10 and 30 kPa) have also been linked to the process of endothelial stabilization and vascular network maturation *via* the increased clustering of integrin and VEGF-responsive signaling. In addition to the elasticity, the matrix viscoelasticity and stress-relaxation kinetics act independently to control the pathways of mechanosensitivity. Stereo-adaptable, rapidly stress-relaxing hydrogels allow greater cellular spreading, cytoskeletal remodelling and YAP/TAZ activation despite identical initial elastic moduli, permitting more matrix deposition and stem-cell differentiation (Chaudhuri et al., 2020). Likewise, the density of ligand presentation regulates integrin occupancy and amplitude of the downstream FAK/MAPK pathway, and degradation kinetics dynamically regulate nutrient diffusion, accessibility of growth factors and temporal persistence of signaling gradients. Together, these experimentally validated relationships inform the idea that bioinks can be utilized as active systems-level perturbation platforms that can rewire intracellular regulatory networks in a quantitatively controllable way. Within regulatory networks, this rewiring may influence lineage commitment, proliferation potential, or stress tolerance in bioinks by reorienting cells toward different attractor states.

In order to evaluate baseline and bioink-exposed cellular state, multi-omics integration and network modelling can be used to quantitatively examine pathway rewiring from a systems biology perspective. The differential activation of cytoskeletal, metabolic and inflammatory pathways has been demonstrated through transcriptomic and proteomic analyses of cells cultured on substrates with defined mechanical properties (Soundararajan et al., 2022). This work demonstrates the propagation of microenvironmental modifications across molecular layers. By uncovering modules whose connectivity patterns are changed by material inputs, network-based techniques like differential network analysis and pathway enrichment mapping represent emergent attributes (Elango and Zamora-Ledezma, 2025) that are not visible through single-gene analysis. Integrating bioink engineering with such systems-level frameworks provides a predictive technique for rationally engineering materials that direct preferred cellular phenotypes *via* controlled signalling network reconfiguration.

### 3.2 Dynamic and mechanistic modeling

#### 3.2.1 ODEs, agent-based models, hybrid mechanochemical models

Dynamic and mechanistic modelling provide a quantitative foundation to comprehend how cells respond to bioink-derived stimuli throughout time (Elango and Zamora-Ledezma, 2025). In systems biology, models based on ordinary differential equations (ODEs) have been extensively used to anticipate how perturbations spread through molecular networks by characterising the temporal dynamics of signalling cascades, gene feedback loops and regulatory circuits (Glass et al., 2021; Hsiao and Dutta, 2024). ODE models can mimic mechanotransduction pathways (such as integrin-FAK-MAPK or YAP/TAZ signalling) that are triggered by matrix stiffness or ligand density in the context of bioinks (Liu et al., 2015; Tiskratok et al., 2025), connecting material characteristics to transcriptional programs downstream. In addition to population-level dynamics, agent-based models (ABMs) capture heterogeneity by encompassing each cell as autonomous agents that respond to local gradients, matrix architecture, and neighbouring cells (Glen et al., 2019). This approach is frequently employed in the modelling of cancer systems and tissue morphogenesis. These frameworks are especially pertinent to 3D bioprinted designs because emergent tissue organization is significantly influenced by spatial heterogeneity and cell-matrix reciprocity.

#### 3.2.2 Capturing temporal evolution of cell states in bioinks

Hybrid mechanochemical models extend this paradigm a step further by combining biochemical signalling networks with mechanical forces and matrix remodelling, allowing cytoskeletal tension, stress distribution and molecular feedback to be described simultaneously (Copos and Mogilner, 2020; Rashid et al., 2025). In bioinks, where early mechanosensitive signalling events can gradually reconfigure transcriptional and metabolic networks to promote proliferation, differentiation, or quiescence (Shou et al., 2023), such multi-scale techniques are crucial for documenting the temporal evolution of cell states. Systems biology makes it possible to identify crucial control nodes that regulate phenotype stabilisation in artificial microenvironments and forecast state transitions by combining dynamic simulations with time-resolved omics data. A conceptual framework for refining bioink compositions to direct specified cellular trajectories across therapeutically significant time scales is provided by this modeling-guided approach.

### 3.3 Machine learning and predictive modeling

#### 3.3.1 Multi-task learning for multiple biological outcomes

To predict cellular responses in artificial microenvironments, machine learning (ML) has developed as a powerful systems-level tool for combining high-dimensional biological and material data (Wang et al., 2026). In bioink research, physical characteristics (including stiffness, porosity, and crosslinking density) as well as transcriptomic, proteomic, and phenotypic readouts can be integrated into machine learning models to comprehend nonlinear interactions that control decisions regarding cell destiny (Sari et al., 2022). Multi-task learning (MTL) enables the simultaneous prediction of many biological outcomes, including cell viability, lineage commitment, proliferation, and matrix deposition (Wu et al., 2024), by leveraging common representations across related tasks. In biomedical applications, MTL enhances performance in multi-phenotype prediction and multi-omics data integration (Eissa et al., 2024). Compared to training individual models for each endpoint, this method improves generalisation and resilience. Predictive modelling can be in line with the essentially multifaceted nature of cell-material interactions in the setting of bioinks by utilising MTL frameworks to capture coordinated regulatory processes that link mechanical stimuli to.

#### 3.3.2 Technical considerations and limitations in AI/ML-driven biofabrication

Although the use of artificial intelligence (AI) and machine learning (ML) is increasingly used in predictive biofabrication, some technical constraints are not eliminated. Strong predictive modeling involves big, regular, and multimodal datasets that incorporate rheology data, imaging data, printing data, transcriptomics, proteomics, and longitudinal biological data. Nevertheless, biofabrication datasets are often confined by small sample sizes, batch effects, inter-laboratory variability, and varied reporting standards, which raise the chances of overfitting the model and decreasing the generalizability. The problem of feature engineering is of high importance as biologically relevant features are to be derived based on heterogeneous data modalities, such as scaffold mechanics, spatial imaging, and network signatures of omics. Graph neural networks, latent-space embedding, and attention-based architectures are becoming popular multimodal integration strategies that can be used to integrate these different datasets into single predictive models. Additionally, stringent validation plans such as cross-validation, external benchmarking, and independent experimental replication are critical in order to provide model robustness and translational reliability. Interpretability of models is also especially significant in biomedical applications where explainable AI methods are needed to determine biologically meaningful correlations between bioink parameters and cellular responses, and not to use black-box prediction alone. These challenges should be addressed to build clinically reliable and reproducible AI-assisted predictive biofabrication systems.

Combined, these systems biology, multi-omics, and computational modeling models provide the basis of integrating bioink physicochemical properties and cellular reactions on a network scale, as described in the next section.

Together, these computational and systems-level strategies offer the methodology that is needed to mechanistically relate bioink properties to pathway rewiring, emergent cellular phenotypes and tissue-level outcomes in predictive biofabrication systems.

## 4 Integrating bioink design with cellular network modelling

### 4.1 Mapping bioink parameters to network-level responses

Bioink features including stiffness, degradability and ligand density had a direct impact on intracellular signalling networks *via* receptor-based and mechanotransduction pathways (Abdel Fattah and Ranga, 2020; Jeong and Lee, 2026). In particular, matrix stiffness modifies transcriptional programs that regulate cell growth and lineage specification by regulating integrin clustering and downstream YAP/TAZ nuclear localisation (Ishihara and Haga, 2022). Similarly, ligand density affects receptor occupancy and pathway amplitude, while degradability modifies matrix remodelling and growth factor accessibility, which alters the signalling gradients and metabolic processes (Tito et al., 2025). Quantitative analyses also show that the mechanotransduction responses induced by bioink are very parameter-specific and time-varying. Indicatively, higher crosslinked GelMA matrices (>50,150 kPa) have been linked to elevated YAP/TAZ nuclear translocation, augmented actomyosin contractility, and augmented osteogenic differentiation indicators such as RUNX2, osteopontin, and alkaline phosphatase activity. Conversely, softer, and more compliant hydrogels (∼2–10 kPa) are conducive to reduced cytoskeletal tension, reduced RhoA/ROCK signaling, and improved neurogenic or pro-angiogenic phenotypes. Matrix mechanics also have a strong influence on macrophage polarization with softer viscoelastic environments only favoring anti-inflammatory M2-like phenotypes, whereas rigid matrices induce inflammatory M1-associated transcriptional programs. The kinetics of degradation also control the activation of temporal pathways by controlling matrix remodeling and long-term release of encased cytokines or growth factors. Matrices with rapid degradation enhance vascular infiltration and tissue remodelling at the cost of structural stability in the long-term, whereas slower degrading systems extend mechanical signalling and the maintenance of focal adhesions. All these observations are consistent with the view that bioink parameters are quantitative controllers of pathway rewiring, which allow programmable control of emergent cellular states and tissue-level organization. Through systematically mapping these material properties to route-level activation patterns, systems biology techniques such as differential network analysis and pathway enrichment modelling render it possible to identify important regulatory nodes within gene-protein-metabolite networks.

Nowadays, perturbation-driven modelling and causal inference frameworks are being employed to separate correlation from fundamental causes in cell–material interactions (Gavriilidis et al., 2024), going beyond empirical correlations. Adapted to bioink research, Bayesian network reconstruction and intervention modelling can be used to predict how specific changes in stiffness or ligand presentation causally rearrange signalling circuits (Tong et al., 2026). These techniques are commonly employed in systems biology to infer regulatory hierarchies. By determining which pathways are changed as well as which material factors have upstream regulatory control over network state transitions, such integration aids in the design of rational bioinks.

### 4.2 Data fusion across scales

Bioink systems are the combination of integrative modeling including single-cell omics, high-resolution imaging, quantitative material properties, and the merging of multi-scale datasets. Proteomics and single-cell RNA sequencing provide high-dimensional mapping of biological states, whereas imaging records matrix architecture, cell shape, and spatial organization (Gulati et al., 2025). Systems-level understanding of how material stimuli modify molecular modules is made easier by network-based integration frameworks that allow omics characteristics to be embedded onto interaction graphs generated from curated route databases (Kant et al., 2025). The integration of biological network reconstruction and materials science measurements allows for the prediction mapping of emergent phenotypes to artificial microenvironments.

By collecting gene expression in its natural spatial context, spatial omics technologies provide a vital link between structure and function, connecting bioink architecture to localised signalling responses (Liu L. et al., 2024; Lázár and Lundeberg, 2026). Spatially resolved transcriptomics and proteomics enable the detection of region-specific pathway activation patterns in 3D bioprinted structures, which may have gradients of stiffness or nutrition transport. The way that structural heterogeneity promotes functional divergence across the construct is more accurately represented when these data are incorporated into graph-based or mechanochemical models.

### 4.3 Integrated systems biology framework for predictive biofabrication

One of the key specifications of predictive biofabrication is the intentional synthesis of bioink physicochemical characteristics, multi-modal biological information and computational design systems into a coherent systems-level structure. Bioinks in this regard can be considered as both structural biomaterials and dynamic perturbation platforms that can regulate intracellular signaling, mechanotransduction, metabolic and spatial cellular organization. The material properties such as stiffness, viscoelasticity, degradability, ligand density and bioactive factor presentation can result in biologic responses, which can be detected using transcriptomics, proteomics, metabolomics, imaging, rheological profiling and spatial omics technologies.

Such heterogeneous data may then be combined using network biology methods, mechanistic models and machine learning systems to discover rewiring events and new cellular phenotypes in pathways. Mechanotransduction signaling pathways that include integrin/FAK, RhoA/ROCK, MAPK, and YAP/TAZ signaling offer key interfaces between extracellular bioink characteristics and intracellular transcriptional and metabolic programs. At the same time, single cell and spatial omics technologies allow a comprehensive high-resolution characterization of cellular heterogeneity, lineage patterns and localized signaling niches in biofabricated environments.

The use of AI/ML-assisted predictive modeling also facilitates multimodal integration of data, feature extraction, and prediction of the outcome through correlation between fabrication parameters and biological reactions such as osteogenesis, vascular maturation, immune modulation, and tissue stability. Notably, predictive biofabrication is based on feedback loops where experimental-validated biological results lead to the successive optimization of bioink design, computational models, and fabrication parameters. This systems-level integration platform transforms biofabrication into an empirically optimized process, to a rationally, predictively, and programmably engineered biomaterial.

### 4.4 Case studies

Integrative modelling techniques can increase predictive accuracy when connecting material qualities to biological outcomes, according to evidence from tissue engineering and mechanobiology. For instance, by identifying YAP/TAZ-centered regulatory modules as mechanosensitive hubs, the combination of transcriptome network analysis with substrate stiffness measurements has improved the prediction of stem cell lineage commitment (Virdi and Pethe, 2021). Similarly, coordinated metabolic and transcriptional rewiring that would not have been discovered by single-endpoint experiments, but has been discovered using multi-omics integration in biomaterial-based differentiation systems. These instances demonstrate that network-guided design solutions surpass solely empirical optimization.

However, there are drawbacks to integrative modelling as well, especially when dealing with sparse, noisy, or temporally misaligned datasets. Overfitting in machine learning models, inadequate mechanical characterisation, or poor sampling across time points can produce erroneous relationships instead of predictions with biological significance (Alber et al., 2019). Furthermore, reliable network signatures may be obscured by batch-to-batch variations in bioink production and biological diversity among cell sources. These challenges highlight the necessity for standardised data collection, multi-objective validation, and hybrid mechanistic-statistical modelling to guarantee that integrative frameworks consistently result in enhanced bioink design and functional tissue engineering outcomes.

System-level and mechanistic approaches capture generalized behaviour of networks, whereas singlecell and spatial omics approaches have the high-resolution biological context to resolve cellular heterogeneity within biofabricated environments.

## 5 Single-cell and spatial omics in bioink systems

The integration of single-cell and spatial omics within bioink systems has shifted tissue engineering from descriptive morphology toward a mechanistic, spatiotemporally resolved systems biology (Yang et al., 2025). Bioinks act as active biological variables that impose critical mechanical and spatial constraints, acting as bridges between synthetic structures and host cellular environments (Li et al., 2023). The “Biomaterial-mediated Cell Atlas” (BCA) framework leverages high-resolution transcriptomics and epigenomics to characterize how bioink properties including stiffness, ligand density, and viscoelasticity mediate cellular responses at a multi-omics level (Othman et al., 2018).

Single-cell RNA sequencing (scRNA-seq) facilitates the identification of cellular heterogeneity and transitional states within bioprinted scaffolds, overcoming the averaging effects of traditional bulk analysis (Zhou et al., 2024). Experimental evidence indicates that bioink crosslinking density directly steers macrophage polarization; stiffer environments upregulate inflammatory subpopulations, while softer bioinks promote pro-regenerative phenotypes (Khozyainova et al., 2023). Computational approaches like pseudotime and RNA velocity analyses reconstruct differentiation trajectories, revealing how bioink-mediated cues selectively recruit specific cells, such as CD93^+^ endothelial progenitors to accelerate graft integration (Liu X. et al., 2024).

Spatially resolved transcriptomics and epigenomics anchor molecular data to native tissue coordinates, preserving the positional cues and microenvironmental interactions essential for tissue patterning (Yang et al., 2023). Technologies like sciMAP-ATAC and Stereo-seq map gene expression and chromatin accessibility within tissue microenvironments, identifying specialized niches such as fibrocartilage-like cells ideal for intervertebral disc repair. This integrated approach enables the inference of localized cell-cell communication networks, such as the Jag1–Notch2 signaling axis localized precisely around implanted biomaterials (Shukla et al., 2022).

In clinical oncology, patient-derived tumor-on-chip platforms bioprint patient-specific cells into perfusable microfluidic environments to recreate the complex tumor microenvironment. These systems simulate dynamic variables like nutrient gradients and oxygen levels, recapitulating patient-specific transcriptional profiles predictive of survival and drug resistance. The future direction of the field involves the transition toward AI-driven “intelligent life atlases” and patient-specific digital twins to simulate biomaterial performance in virtual environments (Basu et al., 2022).

**FIGURE 4:**
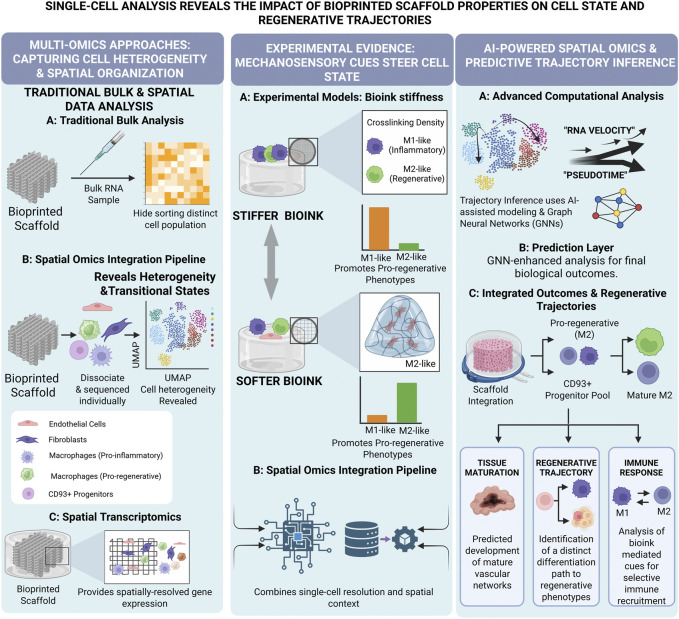
Single cell analysis reveals the impact of bioprinted scaffold properties on cell state and regenerative trajectories. The spatial and single-cell omics techniques can be used to characterize bioink properties and cellular states, immune phenotypes, and regenerative processes in biofabricated systems with exquisite resolution. **(A)** Conventional bulk RNA methods tend to obscure the heterogeneity of cells in bioprinted scaffolds, but single-cell sequencing and spatial omics methods can identify specific cell groups, intermediate phenotypes, and spatially resolved gene-expression profiles. Experimental evidences. **(B)** The stiffness of bioink and crosslinking density regulates mechanosensory signaling and controls macrophage polarization to inflammatory (M1-like) or pro-regenerative (M2-like) phenotypes. Combination of single-cell and spatial datasets can additionally be used to simultaneously analyze cellular identity and tissue-context organization. **(C)** Spatial omics prediction and graph-based trajectory inferences (such as RNA velocity and pseudotime analysis) are assisted by AI to predict regenerative trajectories, tissue maturation, immune responses, and lineage progression in engineered tissues. These strategies together will facilitate predictive biofabrication by correlating scaffold behavior with dynamic states of cell behavior and tissue-scale behavior.

The resolution of cellular heterogeneity and the identification of emergent cell states within bio-fabricated environments mark a fundamental shift from descriptive morphology to a mechanistic systems biology of tissue engineering. Traditional bulk analysis methods rely on averaged signals from mixed cell populations, which inherently obscure critical variations in gene expression and the presence of rare, transitionary cell phenotypes. The Biomaterial-mediated Cell Atlas (BCA) framework addresses this limitation by leveraging single-cell RNA sequencing (scRNA-seq) to provide a granular view of distinct cell types and functional pathways induced by specific bioink properties (Ingber, 2022) (Figure 4).

### 5.1 Mechanistic decoding of bioink-induced heterogeneity

It has been proved experimentally that bioink parameters, including stiffness and crosslinking density, are the major instructive signals of cellular state transitions (Qian et al., 2023). scRNA -sequencing in gelatin methacrylate (GelMA) systems has identified six major macrophage subpopulations, where more rigid, highly crosslinked matrices tumefacilitated inflammatory (M1, M4) and soft bioinks induced pro-regenerative, antigen-processing (M5). Moreover, single cell analysis has played a major role in isolating specialized odontogenic subgroups (marked by SOX4 and IGFBP5) within 3D hydrogel microspheres, thus guaranteeing maintenance of native cellular diversity needed to achieve successful pulp regeneration of the dentin (Dai et al., 2022).

Computational tools that can reconstruct cellular dynamics on the basis of static transcriptomic snapshots are an important contribution to the identification of emergent cell states (Figure 3). There is pseudotime analysis where cells are ordered using virtual timelines to model temporal transitions and has been used to explain the recruitment and late differentiation of endothelial progenitor cells (EPCs) in 3D vascular grafts, thus avoiding thrombosis. In line with this, RNA velocity predictive modeling forecasts future cell states by determining the ratio between unspliced and spliced mRNA and enables the detection of lineage bifurcations in skin wound healing models between profibrotic and regenerative fibroblast states. These observations can be used to give conceptual clarity in the bioink designs that can selectively activate regenerative responses (Groen et al., 2016). Spatial omics, trajectory inference, and AI-aided network analysis integration in biofabricated systems are demonstrated in Figure 4.

Though single-cell dissociation offers excellent molecular profiling, the dissociation of cells causes the blurring of the tissue patterning clues and those cell-cell communication structures necessary to morphogenesis. Spatially resolved transcriptomics (ST-seq) is able to overcome this by mapping molecular information to native coordinates and addressing how positional context in a 3D scaffold determines cellular identity (Zhang et al., 2023). Bone regeneration models that have employed integrated mapping have discovered that there are specialized sub-clusters of mesenchymal stem cell (MSC) clones highly enriched in the matrix Gla protein (Mgp) that are strongly spatially specific to defect sites compared to normal bone (Zhang et al., 2022). Likewise, the architecture of intervertebral disc repair has been solved by means of spatial profiling, which has revealed fibrocartilage-like cells that can be used to reconstruct the intervertebral disc and serve to design inductive composite bioinks (Hall et al., 2020).

With these multi-omic developments, it is confirmed that cellular responses in bioinks are never single occurrences but are spatially orchestrated reactions of localized signaling centres triggered by localised signalling cascades, including the Jag1-Notch2 axis that have been proven around implanted scaffolds (Wang et al., 2025). The final clinical outcome should be the move to the state of intelligent life atlases, where artificial intelligence and machine learning merge real-time multi-omics with high-content imaging to forecast emergent tissue behaviors (Vining and Mooney, 2017). This convergence of bioengineering with the spatiotemporally resolved systems biology forms a basis of resource in the rational design of the next-generation, patient specific cell-instructional biomaterials. The integration of single-cell sequencing, spatial omics, and AI-assisted trajectory inference for decoding bioink-mediated cellular heterogeneity is summarized in Figure 4.

### 5.2 Spatial constraints imposed by bioinks as biological variables

There has been a shift in the conceptual paradigm of tissue engineering where bioinks are no longer considered as passive structural supports but are now seen as active biological variables with a powerful side effect on the spatial and mechanical constraints in cellular behavior. In the Biomaterial-mediated Cell Atlas (BCA) framework, these constraints serve as primary instructive cues that determine cell fate, immunoregulatory phenotypes, and developmental emergent phenotypes (Engler et al., 2006).

Mechanical constraints, namely, stiffness and crosslinking density are the basic variables, which drive the cellular states. Gelatin methacrylate (GelMA) systems have been demonstrated to be facilitated by highly crosslinked and stiff conditions (150 kPa) in stimulating inflammatory subpopulations of macrophages (M1, M4). Soft, and lightly crosslinked bioinks (3 kPa) on the other hand confer the spatial latitude required to induce pro-regenerative, antigen-processing phenotypes (M5) (Chaudhuri et al., 2020). These mechanically generated cellular responses can be mediated by mechanotransduction and pathway rewiring processes that were mentioned in Sections 3.1 and 4.1.

Mechanical constraints are temporal variables in biology since all active tissues are viscoelastic in nature. Bioinks that are designed to develop with rapid stress-relaxation properties have a major impact in bone regeneration as it enables cells encapsulated to remodel their surrounding matrix more effectively. This mechanical memory and the capacity of cells to push or stretch against the cells in a yielding substrate is the direct cause of proliferation and osteogenic differentiation (Alsberg et al., 2001).

Pores, robotically aligned fibers, and curvature of pores make up the structure architecture of a bioink, which forms specific niches that dictate cellular identity (Loh and Choong, 2013). One such biological variable is porosity in which the pore sizes must be suitable and fit to vascularize and induce bone marrow stromal cell differentiation. The existence of these positional cues having an impact on cellular activity has been confirmed by spatially resolved transcriptomics (ST-seq), which revealed that specialized sub-clusters of mesenchymal stem cell (Mgphi) are highly spatially selective to defect sites rather than to mature tissue within a scaffold.

The shear stress and viscosity are biological variables that are brought by the bioprinting process itself. Bioinks with high viscosity (>10,000 mPa s), which are needed to maintain structural fidelity, can be highly restrictive of cellular metabolic activity. Moreover, inappropriate shear stress in extrusion may tear the membranes of cells to release intracellular proteins that become damage-associated molecular patterns (DAMPs) to induce pro-inflammatory conditions. The bioink systems are employed in clinical oncology to duplicate the intricate spatial restrictions of the tumor microenvironment (TME) (El-Kenawi et al., 2020). Tumor-on-chip (TOC) systems bio-print patient-derived cells into configurations which recapitulate native tumor architecture, and model important variables such as nutrient gradients and oxygen concentrations. It has been shown by clinical models of glioblastoma that bioprinted constructs containing these spatial constraints recap patient-specific transcriptional profiles indicative of survival and drug resistance (Xu et al., 2024).

This mechanistic knowledge of the constraints exercised by bioink enables the intelligent design of bioink-based materials that do not just support, but actually direct, the cells to desired therapeutic signatures.

### 5.3 Network inference under spatial dependency

It is a fundamental development in the systems biology of bioink environments to develop methods that, going beyond the observation of discrete clusters of cells, can infer complex regulatory networks that explicitly satisfy the geometrical constraints of space (Levine et al., 2023). In modern computational models, such as the instance of Spatial2Sentence, cellular profiles are redefined as a lexicon, with the spatial coordinates and transcriptional signature as lexical tokens that are read in a more complex, context-sensitive grammar. These models produce the positive pairs of spatially adjacent and transcriptionally consistent cells, which form expression similarity and spatial distance matrices and thus allow large language models (LLMs) to mechanistically learn proximity-based regulatory relationships.

This multi-sentence approach overcomes the inherent limitation of cell culture research to consider cells in isolation, which makes available a more detailed view of the conversation of cells that occurs in the scaffold of a 3D-bioprinted construct (Rao et al., 2021). Complex disease models based on empirical evidence show that incorporation of spatial dependency significantly improves predictive accuracy, improving cell-type classification by nearly six percent and clinical status prediction by more than four percent on top of non-spatial baselines (Abdelaal et al., 2019).

Mechanically, the incorporation of spatial context enables the deduction of localized signalling hubs, e.g., the Jag1-Notch2 axis whose signalling range and functional effectiveness are highly controlled by nearness to the bioink interface. These inferred spatially dependent networks in the clinical oncology arena uncouple organ-specific colonisation in bone-metastatic niches that otherwise remain hidden in non-spatial models of treatment resistance, including metabolic reprogramming. Finally, the future of AI-assisted, spatially sensitive network inference leads to the development of patient-specific digital twins, which can predict the behaviour of physical perturbations within the bioink milieu and bring emergent tissue-level behaviours to life (Loew and Schaff, 2001).

The complexity and dimensionality of the datasets are growing and further highlight reproducibility, harmonization of the data and standard analytic frameworks for predictive biofabrication.

## 6 Managing heterogeneity, batch effects, and reproducibility

The strong connection between bioinks and systems biology, as well as predictive biofabrication, is limited by a high level of heterogeneity among batches of materials, hardware, and biological sources (Velu et al., 2025). Such batch effects should be controlled in order that we can shift the field out of the realm of empirical trial and error to the realm of a reliable and clinically significant discipline (Zhang et al., 2025). The subsequent subsections summarize the key technical, material, and biological sources of variability that together determine the reproducibility, the predictive modeling accuracy and the translational reliability of biofabrication systems.

Another major obstacle to standardisation of bioink production is that performance using natural polymers including collagen and alginate can often be different by 15 0–30 0 -percent lot-to-lot (Ma et al., 2024). With popular materials, like gelatin methacryloyl (GelMA), mechanical properties and the level of functionalisation (DoF) vary dramatically with vendor, batch and internal synthetic control, like stirring rate, and temperature (R Komosa et al., 2024). The reported values of DoF have been as low as 70% and as high as 99% even with the use of the same protocols, resulting in unstable scaffold stiffness and media uptake (Peyret et al., 2023). This variability is material-based and thus a direct effect on predictive data is the capability of the sensitive cellular network models (Young et al., 2020).

Variation in operations, especially at the stage of bioink preparation, brings significant cumulative error (Karvinen and Kellomäki, 2023). The manual mixing methods, i.e., the use of a spatula or the manual dual-syringe pumping, are very reliant on the operator and his or her strength, leading to the non-homogenous cell distribution and the varying viability (Thayer et al., 2018). To overcome these human-caused batch effects, automated active mixing platforms (AAMP) and dedicated specialised mixers including the so-called HighVisc unit have been created to achieve reproducible homogeneity and better cell survival among experimental groups (Li et al., 2024). In addition, pneumatic bioprinters are vulnerable to pressure fluctuations leading to over- or under-extrusion of support-bath qualities implying that standardised reporting of support-bath properties and flow sensor application should be used to enable inter-laboratory reproducibility (Kesti et al., 2016).

Biological inputs, especially induced pluripotent stem cells (iPSCs), are extremely sensitive to the mechanical conditions of the bioink (Ohgushi et al., 2010). The final products of *in situ* expansion and differentiation frequently differ among individual lots of materials as singularised iPSCs are subject to cell death caused by dissociation and have a preferred scaffold stiffness to grow well. Further complicated, there is donor-to-donor heterogeneity in primary cells and variability in donor tissues of decellularised extracellular matrix (dECM) sources (Kim, 2023).

To eliminate them, the direction of the field is moving towards AI-based self-driving laboratories, which combine real-time sensing, safe bioink printing and closed feedback loop technologies. Machine-learning algorithms are also being used to optimise the printing parameters and predict the behaviour of the material which has resulted in the reduction of the optimisation time by up to 40 per cent. Standardising bioink properties on an international level; that is, rheology, gelation dynamics and minimum viability levels is the most important step towards the actualisation of the biofabrication potential.

### 6.1 Variability across labs, printers, cell sources

The integration of bioinks into systems biology and predictive biofabrication is still burdened with the lack of significant consistency of differences in laboratories, hardware platforms and biological inputs. This variability compromises the reproducibility of mechanistic data and interferes with the clinical translation of engineered tissues.

Inadequate standardization of protocols of characterization has led to large inter-laboratory variations. In natural polymers like collagen and alginate, batch-to-batch variation in performance can be 15%–30% due to variation in rheological and gelation characteristics. In more standardized materials, as it is the case with gelatin methacryloyl (GelMA), storage modulus and mechanical integrity vary significantly, depending on the vendor, lot, and internal synthesis requirements, including stirring speed, temperature, and the degree of functionalization thereby affecting directly cellular behavior and experimental accuracy (Liu et al., 2026).

The choice of hardware also brings in additional cumulative errors during the biofabrication process (Rodríguez Rego et al., 2025). Significant inter-laboratory errors in print fidelity remain in print fidelity with the same bioinks and digital models used. Pneumatic bioprinters in particular are prone to instabilities of pressure which can cause over- or under-extrusion thereby affecting construct resolution and mechanical stability. Furthermore, the porosity and stiffness of bioprinted scaffolds may be fundamentally modified by the nature of the support bath such as particle size and yield stress compared to conventional casting (Grijalva Garces et al., 2024).

One of the main points of variability is the biological identity of cellular inputs. Primary cells are constrained by heterogeneity of the donors-to-donor, the native variations in phenotype, and limited proliferative ability (Dufour et al., 2024). The induced pluripotent stem cells (iPSCs) have more versatility, but their survival and growth are very sensitive to the mechanical conditions of the bioink (Xu et al., 2015). The results of *in situ* expansion and differentiation often vary with lots of GelMA, with singleized hiPSCs dissociating and dying as well as being sensitive to scaffold stiffness in order to grow well. It is important to deal with these bottlenecks by developing standardized, autonomous systems to achieve real predictive biofabrication (Mota et al., 2020).

### 6.2 Harmonization strategies for biofabrication datasets

The transition from empirical biofabrication to predictive systems biology requires a robust framework for harmonizing high-dimensional datasets. Current efforts are frequently undermined by a lack of end-to-end standardization in data formats, communication protocols, and quality metrics.

Dataset harmonization begins with the implementation of rigorous reporting standards that extend beyond simple material concentrations. To achieve inter-laboratory interoperability, datasets must capture granular metadata, including the degree of functionalization (DoF), storage/loss moduli, and vendor-specific lot numbers, which fundamentally dictate cellular outcomes (Peyret et al., 2023). Establishing a global consensus on protocols for bioink characterization, specifically rheology and gelation kinetics, is essential to reduce the current 15%–30% performance variability observed between batches (Siwach et al., 2025).

Artificial intelligence serves as a critical tool for integrating multi-modal data from disparate sources. Advanced machine learning models can process multi-dimensional inputs and converge labels across inconsistent experimental conditions, enabling “backward design” where desired biological responses are mapped to specific material architectures (Singh et al., 2026). Furthermore, the emerging discipline of “AM Informatics” manages the lifecycle of additive manufacturing data, preserving the mechanistic relationships between part geometry, material properties, and specific fabrication parameters (Knapp et al., 2017).

To address the scarcity of well-labeled experimental datasets, researchers are increasingly utilizing simulated data from Monte Carlo and molecular dynamics simulations. These simulations generate harmonized libraries for training algorithms in parameters that are physically difficult to characterize, such as polymer permeability and internal shear distributions. These harmonized pools feed into “digital twins,” allowing for accurate predictive modeling of spatial and temporal variations during the printing process (Roth et al., 2018).

The realization of predictive biofabrication necessitates a centralized digital ecosystem. Physical and digital interconnection *via* cloud-based infrastructures enables the seamless transfer of imaging data into bioprinting instructions (G-code) across different hardware platforms (Sexton et al., 2025). Such interoperable systems support real-time sensing and closed-loop feedback, allowing for autonomous error correction and high-throughput production with minimal manual intervention.

## 7 Federated and privacy-preserving frameworks for biofabrication data

Predictive biofabrication is inherently reliant on the incorporation of large, multi-institutional databank, which decouples biomaterial design variables with longitudinal cellular responses. In the context of systems biology, the transition between small lab projects and the enterprise-wide infrastructures is essential to creating a common ontology and standard vocabulary across the dissimilar repositories. These models, including the example of the UI BioShare service, use project management and community-based decisions to align clinical and molecular data to improve access to high-quality, annotated biospecimens to provide predictive modelling. Data veracity and systematic backups required to sustain the integrity of information related to the specimen are ensured by centralized governance using secure servers and assigned roles, which is a critical aspect of large-scale translational research (Santillan et al., 2025).

To address the privacy and regulatory challenges inherent in the decentralized training of sensitive medical data across jurisdictions, federated learning (FL) has become a formidable approach to decentralized model training. Allowing deep learning to run directly within institutional systems, FL avoids the necessity of raw data aggregation, which significantly reduces the risk of breaches, yet FL makes it possible to get insights based on multi-institutional imaging and molecular data. Modern models like DPResNet combine altered architectures with local differential privacy (DP), using gradient clipping and scaled Gaussian noise to blur the contributions of each individual without loss to the prediction performance of the overall model. This strength is further enhanced by the concept of Secure Multi-Party Computation (SMPC) that the update of individual models is kept confidential by central coordinators during the aggregation process (Fares and Saad, 2024). However, current federated learning (FL) approaches for biofabrication are still limited by the diversity of local data, the quality of the imaging, the annotation process, and the availability of computational resources at different institutions. These factors might create model bias and may decrease model reproducibility. Furthermore, privacy-preserving architectures might introduce a computational overhead and restrict the capabilities of updating models in real-time. Therefore, the standards for harmonized metadata, standardized preprocessing workflows, and comprehensible validation procedures are still crucial for the successful deployment of federated AI systems in the realm of predictive biofabrication.

**FIGURE 5:**
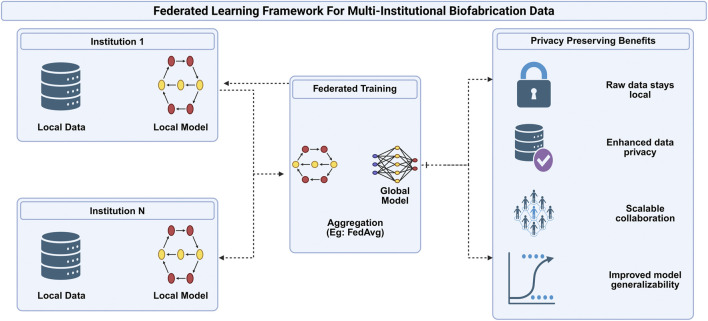
Federated learning framework for multi-institutional biofabrication data. Federated learning architectures enable collaborative AI model training across multiple institutions without centralized sharing of sensitive biological or clinical datasets. Local datasets remain institution-specific while model parameters are iteratively aggregated into a global predictive model through secure federated optimization strategies. These frameworks improve data privacy, scalability, model generalizability, and multi-institutional collaboration while supporting predictive biofabrication and clinically translatable biomaterial design.

In addition to model training *via* iteration, the only systems that are truly privacy-preserving federated analytics, such as FAMHE, use multiparty homomorphic encryption (MHE) to allow the use of complex statistical operations on distributed data without revealing the intermediate values. These frameworks enable precise survival analyses and large-scale genomic association studies which are no different, computationally, than centralized, non-secure methods in terms of accuracy but with encryption security. Infrastructure of this type is especially relevant to biofabrication because it enables the development of unbiased and generalizable clinical recommendations in rare diseases where single-institution cohorts are not sufficiently powerful to provide adequate statistical evidence. Finally, such federated architectures meet the strict legal requirements, including the General Data Protection Regulation (GDPR), as the data manipulated through MHE is virtually anonymized and encourages multi-centric scientific research in predictive biofabrication (Froelicher et al., 2021) (Figure 5)

## 8 Toward predictive and equitable biofabrication

The future of biofabrication is progressively dependent on a smooth fusion of biomaterial design and advanced computational modelling, thus avoiding the need to rely on an iterative experimental trial-and-error paradigm and introducing the discipline to a paradigm of prediction. The modern artificial neural network allows researchers to replicate complex biological processes, carefully testing the parameters (pH, temperature, and concentrations of the substance) to achieve the synthesis of bio-derived nanomaterials with unprecedented accuracy. An archetypal example of using this strategy is demonstrated in the collagen nanoparticles biosynthesis employing a cell-free system that makes use of the *Streptomyces* viridochromogenes. In this framework, machine-learning algorithms outline the optimal conditions of the highest yield and colloidal stability, thus maintaining the structural fidelity requirement to therapeutic bio-ink. These predictive assets are further scaled using large-scale platforms like the Manufacturing Multi-Organs Database that uses regression models to standardize the assessment of material properties and predictive functional measures across a palette of human organ models. This repository is able to provide a finely tuned translation between the engineered constructs and their endogenous biological equivalents, by a systematic correlation of fabrication parameters to organ-specific indicators, e.g., hepatic metabolic secretions. With these systems-biology systems, convergence between the parameters of fabrication and organ-specific biomarkers gradually closes the gap between bioprinted structures and the biofunctions complexity of native tissues. Sustainability and equity are also developed through the use of microbial and marine sources of collagen, which is a biocompatible, environmentally-friendly alternative to standard animal matrices, which also suppresses the risk of immunogenicity and alleviates the problem of cultural sensitivity. Finally, mechanistic modelling of empirical data forms the basis of the dawn of synergistic therapeutics, such as combining colloidal nanoparticles with doxorubicin to obtain potentiated tumouricidal action. These integrative approaches simplify the concept design workflow, reduce resource use, and expedite the biofabrication technology translation to a future of efficiency in operations and standardized clinical implementation (El-Naggar et al., 2025).

## 9 Future direction

The future directions of bioinks within a systems biology framework increasingly depend on integrative, predictive, and multi-scale design techniques. Machine learning (ML) and artificial intelligence (AI) are becoming essential tools for optimising printing parameters, predicting bioink behaviour under different situations, providing real-time process monitoring, and correlating material composition with biological performance outcomes (Pushparaj et al., 2023; Debnath et al., 2025). These data-driven approaches are expected to increase the translational scalability, accuracy, and reproducibility of bioprinting procedures. In order to shed light on cell-matrix interactions, phenotype-microenvironment coupling, and hierarchical scaffold organization across nano-to macro-scales, systems biology principles are being applied to the development of bioinks, particularly collagen-based platforms, using advanced characterisation techniques (Debnath et al., 2025).This systems-level understanding enables the construction of rational microenvironments that mimic natural biochemical signals, including growth factor regulation and integrin interaction (Ullah et al., 2025).

Extracellular vesicle (EV) integration, mRNA-based genetic programming, protein delivery *via* nanoparticles, and microRNA-driven regulation of angiogenic and differentiation pathways are examples of future bioinks that are anticipated to be programmable and functionally adaptive systems(Ullah et al., 2025). These strategies aim to transform bioinks into dynamic cellular signalling network regulators from passive structural matrices. Concurrently, advancements like as AI-enabled 4D bioprinting, automated high-throughput systems, cryogenic printing, and real-time quality control systems are anticipated to eliminate present challenges related to mechanical optimisation, scalability, and clinical translation (Pushparaj et al., 2023). Continued standardization, hybridization of natural and synthetic polymers, and deeper systems-level modeling of cell-material interactions remain essential to fully realize the potential of bioinks as network-modulating platforms in regenerative medicine (Pushparaj et al., 2023; Debnath et al., 2025; Ullah et al., 2025).

## 10 Conclusion

Bioinks can no longer be seen as passive matrices created solely for print quality or short-term viability. In this approach, they are represented as time-varying, multi-scale perturbation systems that dynamically remodel gene regulation, signalling, metabolic, and mechanical networks. The field has been hindered by its persistent dependence on reductionist screening criteria, which put short-term survival ahead of long-term functional maturity and processability ahead of biological durability. Therefore, a systems-level transition is necessary rather than optional.

By integrating multi-omics profiling, spatially resolved transcriptomics, mechanochemical modeling, and multi-task machine learning, bioink research can move from empirical optimization toward causal and predictive design. Network-based modeling enables mapping of stiffness, degradability, ligand density, and architectural constraints to pathway rewiring and phenotype stabilization. Dynamic models further capture temporal evolution of cell states, while harmonized, model-ready datasets mitigate batch effects and inter-laboratory variability.

Future requirements will include interoperable data infrastructures, standardised omics pipelines, and hybrid mechanistic-statistical frameworks that can be applied to a variety of cell sources and manufacturing platforms. When bioinks are integrated into federated and ethically controlled digital ecosystems, they transition from structural carriers to programmable regulators of tissue-level behaviour. To achieve predictive and equitable biofabrication, material engineering and systems biology principles must be applied to generate predictable biological trajectories rather than structures.
